# The Effects of Aroma on Apnea Attacks and Oxygen Saturation Among Preterm Infants: A Systematic Review and Meta-Analysis -

**DOI:** 10.31661/gmj.v12i.2846

**Published:** 2023-05-08

**Authors:** Sanaz Rustaee, Rafat Rezapour-Nasrabad, Masumeh Ghazanfarpour, Saeedeh Piri, Seyedeh Fatemeh Moosavi Moqaddam, Arian Behdarvandi, Fatemeh Kafami Ladani, Ahmad Rahmah, Maryam Mirzaei

**Affiliations:** ^1^ Department of Nursing Internal Surgery, School of Nursing, Fasa University of Medical Sciences, Fasa, Iran; ^2^ Department of Psychiatric Nursing and Management, School of Nursing and Midwifery, Shahid Beheshti University of Medical Sciences, Tehran, Iran; ^3^ Nursing Research Center, Kerman University of Medical Sciences, Kerman, Iran; ^4^ Department of Nursing, Lorestan University of Medical Science, Shahid Madani Hospital, Khorramabad, Iran; ^5^ Department of Nursing, School of Nursing, Jahrom University of Medical Sciences, Jahrom, Iran; ^6^ Department of Biology, Shahid Chamran University of Ahvaz, Ahvaz, Iran; ^7^ Department of Nursing, Nursing Care Research Center, Iran University of Medical Sciences, Tehran, Iran; ^8^ Department of Clinical Pharmacy, College of Pharmacy, Mustansiriyah University, Baghdad, Iraq; ^9^ Department of Obstetrics and Gynecology, Faculty of Medicine, Jiroft University of Medical Sciences, Jiroft, Iran

**Keywords:** Premature, Aromatherapy, Herbal, Infants, Oxygen Saturation

## Abstract

Researchers and clinicians need to be aware of procedures that are more adaptable to new and different environments in premature infants; therefore, it is important to conduct a comprehensive review of the effect of aromatherapy on apnea attacks and oxygen saturation (SpO2 ) in premature newborns. In this review, databases such as PubMed, Scopus, Web of Science, and Cochrane Library were systematically searched without language and time limitations up to November 1, 2022. Initially, 153 studies were founded, and after duplicate removal, title as well as full-text review, seven studies were enrolled in the final analysis. Studies indicated that aromatherapy with Rosa damascena, vanilla, and breast milk odors could significantly reduce apneas in preterm infants and improve SpO2 levels. Hence, aromatherapy could consider as an effective adjuvant treatment for the reduction of apnea attacks among preterm infants.

## Introduction

Providing suitable environmental conditions, especially in high-risk groups, including children, women, the elderly [1][2][3][4], and patients with life-threatening diseases, is one of the most important duties of the medical team [5][6][7][8][9][10]. Meanwhile, premature infants and their mothers are among the most critical groups receiving medical care [1][2][3]. Premature labor (before 37 weeks of gestation) increases the probability of fatality and morbidity due to respiratory immaturity [11].

Apnea-a brief pause for more than 20 seconds-is one of the most prevalent complications experienced by premature infants admitted to the neonatal intensive care unit [12]. In other words, apnea, pallor, bradycardia, and cyanosis [13] are reported in approximately 85% of infants with a gestational age equal to or less than 34 weeks [14]. Also, apnea could lead to brain damage, hypotonia, hypotension, neurological disorders, hydrocephalus, and even mortality [15]. The recurrent apnea and the need for mechanical ventilation within the first week of treatment could be decreased using methylxanthines, such as aminophylline, theophylline, and caffeine [14][16][17]. However, they cannot completely prevent apnea sequels, and additionally, several undesirable side effects such as hyperactivity, irritability, sleep disorders, tachycardia, and urinary disorders have been noticed in treated infants [18]. As a natural treatment, aromatherapy uses aromatic essential oils extracted from various parts of plants, such as leaves, bark, fruits, stems, seeds, roots, and flowers [19]. The olfactory and gustatory receptors develop by the eighth week of gestation and become functional by the 24^th^ and 17^th^ weeks, respectively [20]. Several studies showed the effects of aromatherapy with vanilla [11][15][19][21], *Rosa damascena* [18], and breast milk odor [13][14][15] on apnea attacks and oxygen saturation (SpO_2_ ). Hence, this study aimed to provide evidence through a systematic review of the effects of aromatherapy on apnea attacks and SpO_2_ in preterm infants.

## Materials and Methods

*Search Strategies*

This systematic review was conducted according to the PRISMA 2020 Checklist [22]. A comprehensive literature search was conducted in PubMed, EMBASE (via OVID SP), Scopus, and Web of Science until November 2022. We used the medical subject heading (MESH) and free text words for our search in different combinations, such as ((Preterm OR (Premature) AND (Odor OR Olfactory OR Aroma OR aromatherapy OR Smell OR odor) AND (Apnea OR oxygen saturation OR Spo_2_ ). Search strategies were modified as necessary for each specific database. The full search strategies for each database are presented in Table-1.

All the articles found throughout our search process were imported into Endnote X6 (Thomson Reuters, Philadelphia, PA, USA), and duplicates were removed. The titles and abstracts of identified studies were scrutinized for eligibility by four reviewers (SR, MGH, SP, and SFMM) in two groups independently,and full-text versions of selected studies were collected for further assessment. Four reviewers in two different groups independently examined the full texts, and relevant articles were identified. The discrepancies were examined and finalized by the corresponding author (MM). Also, for better coverage, reference lists of selected articles and review papers were manually searched to identify relevant articles.

**Table:** Table 1. The Search Strategies In The Different Databases

**Database**	**Search statement**
PubMed	((((("Infant, Premature"[Mesh] AND "Infant, Extremely Premature"[Mesh]) AND ( "Infant, Premature/blood"[Mesh] OR "Infant, Premature/cerebrospinal fluid"[Mesh] OR "Infant, Premature/growth and development"[Mesh] OR "Infant, Premature/immunology"[Mesh] OR "Infant, Premature/metabolism"[Mesh] OR "Infant, Premature/physiology"[Mesh] OR "Infant, Premature/psychology"[Mesh] OR "Infant, Premature/urine"[Mesh] )) AND ( "Infant, Extremely Premature/blood"[Mesh] OR "Infant, Extremely Premature/cerebrospinal fluid"[Mesh] OR "Infant, Extremely Premature/growth and development"[Mesh] OR "Infant, Extremely Premature/immunology"[Mesh] OR "Infant, Extremely Premature/metabolism"[Mesh] OR "Infant, Extremely Premature/physiology"[Mesh] OR "Infant, Extremely Premature/psychology"[Mesh] OR "Infant, Extremely Premature/urine"[Mesh] )) AND ( "Aromatherapy/adverse effects"[Mesh] OR "Aromatherapy/classification"[Mesh] OR "Aromatherapy/economics"[Mesh] OR "Aromatherapy/ethics"[Mesh] OR "Aromatherapy/history"[Mesh] OR "Aromatherapy/instrumentation"[Mesh] OR "Aromatherapy/methods"[Mesh] OR "Aromatherapy/mortality"[Mesh] OR "Aromatherapy/nursing"[Mesh] OR "Aromatherapy/psychology"[Mesh] OR "Aromatherapy/standards"[Mesh] OR "Aromatherapy/statistics and numerical data"[Mesh] OR "Aromatherapy/trends"[Mesh] OR "Aromatherapy/veterinary"[Mesh] )) AND ( "Odorants/analysis"[Mesh] OR "Odorants/legislation and jurisprudence"[Mesh] OR "Odorants/prevention and control"[Mesh] )) AND "Agnosia"[Majr]
Web of Science	TI= (Infant * OR "Infant, Premature") AND TI=("Bariatric Surgery" OR "Preterm Infant" OR "Premature Newborn" OR "Preterm Neonate" OR " Prematurity ") AND TI=("Aromatherapy*" OR " Odor " OR Smell *) AND TI=(" Olfactometer *" OR " Olfactory stimulation*")
Scopus	(TITLE-ABS (Infant *) OR TITLE-ABS ("Infant, Premature ")) AND (TITLE-ABS("Preterm Infant") OR TITLE-ABS("Preterm Newborn ") OR TITLE-ABS("Premature Newborn ") OR TITLE-ABS(Premature Neonate) OR TITLE-ABS("Preterm Neonate ")) AND (TITLE-ABS(Prematurity *) OR TITLE-ABS("Aromatherapy ") OR TITLE-ABS(Odor) OR TITLE-ABS(Smell *)) AND (TITLE-ABS(Olfactometer *) OR TITLE-ABS(Olfactory stimulation *))
Cochrane Library	Title Abstract Keyword Premature infant AND Title Abstract Keyword Prematurity AND Title Abstract Keyword odour AND Title Abstract Keyword Olfactory stimulation AND Title Abstract Keyword Preterm infant AND Title Abstract Keyword odor AND Title Abstract Keyword Olfactometer AND Title Abstract Keyword smell Title Abstract Keyword Premature infant OR Title Abstract Keyword Prematurity OR Title Abstract Keyword odour OR Title Abstract Keyword Olfactory stimulation OR Title Abstract Keyword Preterm infant OR Title Abstract Keyword odor OR Title Abstract Keyword Olfactometer OR Title Abstract Keyword smell
Embase	('Preterm infant':ab,ti OR 'Premature infant':ab,ti OR 'Preterm Newborn':ab,ti OR 'Premature Newborn':ab,ti OR ' Premature Neonate':ab,ti OR 'Preterm Neonate':ab,ti OR 'Premature baby':ab,ti OR 'Preterm baby':ab,ti OR ' Preterm infant, Aromatherapy':ab,ti OR ' Preterm infant; Aroma':ab,ti OR ' Preterm infant, Smell ':ab,ti AND 'Aromatherapy':ab,ti OR 'Aroma':ab,ti OR 'Smell':ab,ti OR 'Odor':ab,ti OR 'odour':ab,ti OR 'Olfactometer':ab,ti OR 'Olfactory stimulation':ab,ti)

*Eligibility Criteria and Study Selection*

We included all clinical trials that exanimated the impact of aromatherapy on SpO_2 _and apnea attacks among preterm infants (less than 37 weeks of gestation). Also, non-clinical trials, non-human studies, reviews, letters to the editor, multi-sensorial interventions, conference papers, and non-published data were excluded.

*Data Extractions*

After selecting the relevant studies, data, including first author name, year of publication, type of control, intervention interval, and aroma dosage were entered in a pre-developed form.

*Quality Assessments*

We used the modified Jadad tool, a checklist that evaluated clinical trial studies [9]. Briefly, Jadad consists of eight questions in five domains (e.i., description of randomization, methods used to generate the sequence of randomization, blinding, method of blinding, description of withdrawals, and dropouts) that scored separately. The maximum score obtained from the Jadad is equal to eight. Accordingly, studies were categorized into three groups low-(score<4), moderate- (score 4 to 6), and high- (score≥6) quality. Two authors (SR and MM) independently performed the quality assessment, and a consensus was made in the case of disagreement.

*Statistical Analysis*

The heterogeneity of studies was evaluated by the Chi-squared and I2 tests. The results were reported using a random effects model, and the standardized mean difference (SMD) method was applied to the intergroup comparisons at a 95% confidence interval (CI). Also, the "Metaprop" command was used to perform meta-analyses of proportions on STATA14 (Stata, College Station, TX, USA)

## Results

*1. Characteristics of Studies*

**Figure-1:**
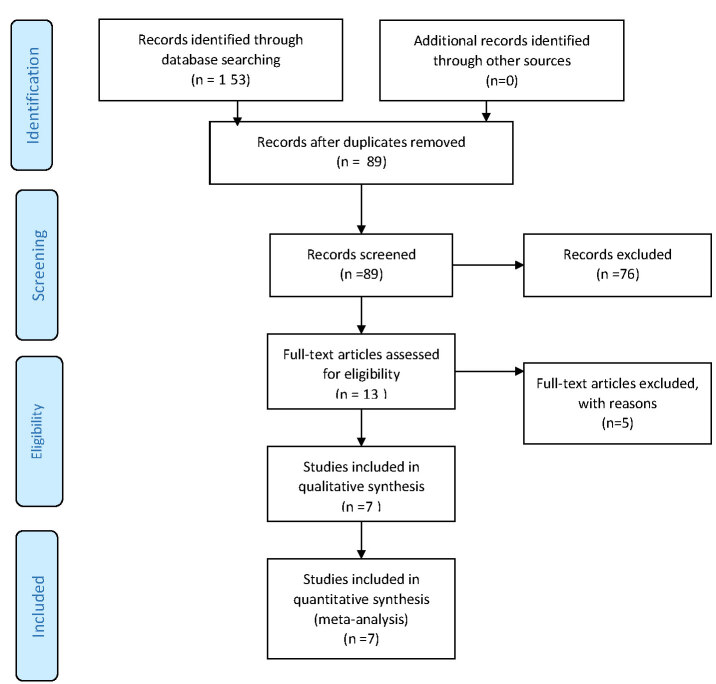
Figure 1-PRISMA flowchart of the study.

Our initial search identified 153 articles, and after duplicate removal, as well as the title and abstract screening, 13 articles were chosen for full-text review. Finally, seven studies [11][15][19][21][23][24][25] were included in our review (Figure-1). The characteristics of included studies are presented in Table-2. Totally, 463 preterm infants were evaluated. Aromatherapy with vanilla, R. damascena, and breast milk odor was performed in four [11][15][19][21], one [25], and four [11][19][23][24] studies, respectively. The Jadad score for included studies are shown in Table-3.

**Table:** Table 2. Characteristics of Included Studies.

**Authors**	**Location**	**Groups/Intervention**	**Dose/Interval**	**Outcome(s)**
Kanbur et al., 2019 (21) Turkey	Turkey	-Breast milk (n=13) -Vanilla (n=16) -Control (n=13)	Was placed in the incubators	A significant decrease in the frequency of apnea in preterm neonates in the vanilla group compared to the breast milk and control
Edraki et al., 2013, Iran(15)	Iran	-Vanillin (n=18) -Control (n=18)	A piece of cotton inside the incubator/ every 12 hours	A significant difference was found between the intervention group and control regarding spo2 (P=0.02) and apnea (P<0.05).
Yaghoubi et al., 2017, Iran (22)	Iran	-Vanilla (n=19) -Control (n=18)	A piece of cotton approximately 20 cm from infants/ every 12 hours	The frequency of apnea attacks and SPO2 level changed after treatment with cotton impregnated with 2ml of vanillin extract (p=0.016) in comparison with the control group
Aghagoli et al., 2016; Iran (26)	Iran	Rosa damascena distillate10% (n=30); Control group (n=30)	-Two drops/ every three hours for three days	The number of apnea attacks and SPO2 levels was significantly lower in the Rosa damascenes group than in control (distilled water) (P< 0.05)
Neshat et al., 2016, Iran (23)	Iran	-Vanilla (n=45) -Breastmilk (n=45) -Control (n=45)	- 10 drops vanilla -Solution placed on cotton five minutes before sampling -Until 30 seconds after sampling	The breast milk odor group significantly benefited premature infants’ SPO2 during (p = 0.014) and after venipuncture (p=0.04). But Changes in blood oxygen saturation were not significant between in the vanilla odor and control group during (p = 0.16) and after (p=0.44) veodor re
Alemdar et al. 2019; Turkey (25)	Turkey	Breast milk odor group n=30, Mother voice group n=30; incubator Cover group n=31; control group n=32	Solution placed on gauze sponge 15 min before Peripheral cannulation. And up to 15 min after the procedure. .	The breast milk odor groups were not different from the mother voice and control group in terms of mean SO2(p>0.05)
Park et al. 2020(24) Korea	Korea	2cc of mother’s breast milk (n=14), 2cc of saline solution (n=16)	Breast milk was placed 10 cm away from the infants/ 8 times per day for 3 consecutive days.	Any significant differences in oxygen saturation levels (p=0.5) between the milk odor group and control groups

**Table:** Table 3. The Quality of Included Studies Based on Jadad

**Questions**	**Edraki et al.**	**Kanbur et al.,**	**Yaghoubi et al.,**	**Neshat et al.,**	**Park et al.**	**Alemdar et al.**	**Aghagoli et al.,**
Was the study descript as randomized?	5	5	5	5	5	5	5
Was the methods of randomization appropriated?	5	4	5	5	5	5	5
Was the study descript as blinded?	5	5	4	5	4	4	4
Was the methods of blinding appropriated?	4	5	4	4	5	5	5
Was a description of withdrawals and dropouts?	4	5	5	4	5	5	5
Was a clear description of inclusion and exclusion criteria	5	5	5	5	5	5	5
Was the method used to assess the adverse effects described?	5	5	4	4	5	5	5
Was the method of statistical analysis described?	5	5	5	5	5	5	5
**Total score**	38	38	37	37	39	39	39

*2. Effect of Aromatherapy On Apnea Attacks*

*2.1. Vanilla*

Although four studies [11][15][19][21] assessed the effect of aromatherapy with vanilla, only three studies [15][19][21] indicated therole of vanilla on the frequency of apnea attacks (Table-2).

Edraki *et a*l. [15] showed that receiving vanilla solution (2%) could significantly reduce apnea attacks compared to the control group (Table-2). Also, Kanbur *et al*. [19] reported a significant decrease in the frequency of apnea in preterm neonates in the vanilla group compared to the breast milk and control groups.In Yaghoubi *et a*l. study [21], the frequency of apnea attacks changed after treatment with cotton impregnated with 2ml of vanilla extract compared to the control group (P=0.016, Table-2). 

*2.2. R. damascena*

In a study by Aghagoli *et al.* [25], the number of apnea attacks was significantly lowered in the *R. damascena* group than in control (P<0.05, Table-2).

*3. Effect of Aromatherapy on SpO_2_*

**Figure-2:**
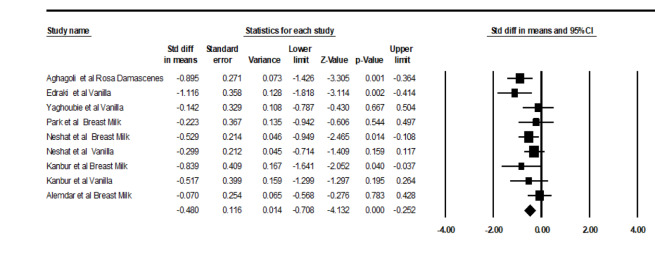
**Figure 2.** The effects of aromatherapy on SpO_2_. The horizontal lines denote the 95% CI, ■ point estimate (size of the square corresponds to its weight); ♦, combined overall effect of treatment

Overall, the SMD of SpO_2 _between the aromatherapy and the control group was -0.48 (95%CI: -0.708 to -0.25, I2 =28.1%), which indicates significant differences (P˂0.001, Figure-2).

*3.1. Vanilla*

**Figure-3:**
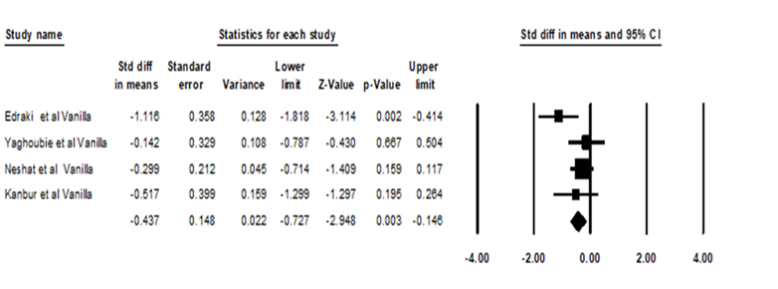
**Figure 3.** The effects of aromatherapy with Vanilla. The horizontal lines denote the 95% CI, ■ point estimate (size of the square corresponds to its weight); ♦, combined overall effect of treatment.

Regarding Figure-3, the SpO_2_ levels of the *vanilla* group were significantly different compared to the control group (SMD: -0.43,95%CI: -0.72 to -0.14, P=0.003; Figure-3).

In Yaghoubi *et al.* study [21], a significant difference was observed in SpO_2 _levels of premature neonates with apnea between the vanilla group and the control (Table-2). Also, Edraki *et al*. [15] reported a significant difference in SpO_2_ between the vanilla group and the control group (P=0.02, Table-2). In contrast, Neshat *et al.* [11] showed no significant differences among preterm infants of vanilla and control groups regarding SpO_2_ (Table-2).

*3.2. R. damascena*

In the study of Aghagoli *et al*. [25], decreased SpO_2_ level was significantly (P<0.05) improved in the 10% *R. damascena* group than in control (distilled water) among preterm infants with apnea (Table-2).

*3.3. Breast Milk*

**Figure-4:**
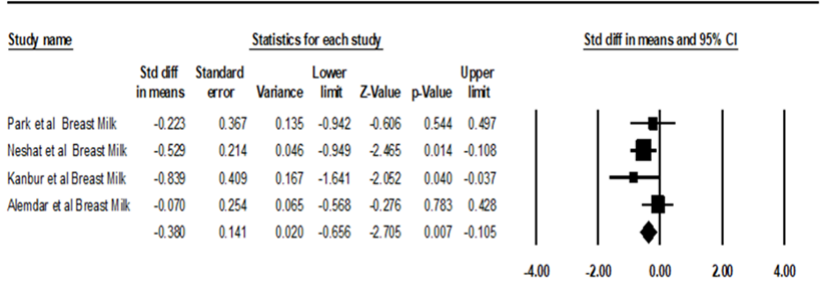
**Figure 4.** The effects of aromatherapy with breast milk odor on SpO_2_. The horizontal lines denote the 95% CI, ■ point estimate (size of the square corresponds to its weight); ♦, combined overall effect of treatment.

The SpO_2 _ levels between breast milk and the control groups were significantly different (SMD=-0.38, 95%CI: -0.66 to -0.105, P=0.007, Figure-4) Park *et al*. [23] demonstrated no significant differences in SpO_2 _ levels between the milk odor and control groups (P=0.548, Table-2). Also, Alemdar *et al*. [24] indicated that the mean level of SpO_2 _was not significantly different in breast milk odor groups compared to mother voice and control groups (Table-2). However, Neshat *et al.* [11] stated that the breast milk odor group could significantly improve the SpO_2_ of premature infants compared to the control group (P=0.014, Table-2).

## Discussion

The current systematic review found that aromatherapy had both preventive [15] and therapeutic effects [18] on apneas in preterm infants. Ataei Nakhaei *et al*. [26] in a systematic review, revealed that aromatherapy effectively treats apnea in preterm infants. However, the authors reiterated that their findings should be interpreted cautiously due to the small sample size and the low number of studies [26]. Evidence indicated that vanilla could impact on apnea in some ways. Indeed, vanilla could be

absorbed via the nasal mucosa and enter the brain through the bloodstream and improve orbitofrontal blood flow [15]. Also, vanilla helps newborns deal with stress and directly affects the respiratory centers [18]. Hence, it balances psychological and physiological states [18]. Aromatherapy with the *R. damascena* could be applied as an effective intervention for premature infants suffering from apnea, followed by routine therapy to reduce bradycardia and improve SpO_2_ levels [25]. The extract of *R. damascena* contains christin and kaempferol, which has some beneficial effects on the nervous system [27] and reduces pain [28]. Moreover, the hydro-alcoholic extract of* R. damascena* has a dilatory effect on respiratory airways [25][28]; hence, it could reduce anxiety as well as improve sleep quality, especially in premature infants hospitalized due to apnea [29][30][31]. Although the current study involved more studies compared to previous research [26], there were some limitations. Indeed, the studies included in our systematic review had a small sample size, and only one type of apnea (i.e., the idiopathic apnea of prematurity) was evaluated. Hence, further investigations to verify the effectiveness of this non-pharmacological method in various types of apnea are recommended. 

## Conclusion

Aromatherapy with natural substances (e.g.,* R. damascena*) and even artificial ones can be used as an additional treatment to improve the breathing condition of premature infants with apnea.

## Conflict of Interest 

Authors declare there were no any conflicts of interest

## References

[R1] Mohammadi F, Tabatabaei HS, Mozafari F, Gillespie M (2020). Caregivers' perception of women's dignity in the delivery room: a qualitative study. Nurs Ethics.

[R2] Mohammadi F, Oshvandi K (2020). Male nursing students' perception of dignity in neonatal intensive care units. Nurs Ethics.

[R3] Mohammadi F, Kiani A, Gholamzadeh S, Asadi Noghabi, Sadeghi T (2018). The factors affecting successful breast-feeding (SBF). Iranian Journal of Neonatology.

[R4] Mohammadi F, Rakhshan M, Molazem Z, Zareh N, Gillespie M (2020). Development of parental competence scale in parents of children with autism. J Pediatr Nurs.

[R5] Tehranineshat B, Mohammadi F, Tazangi RM, Sohrabpour M, Parviniannasab AM, Bijani M (2020). A study of the relationship among burned patients' resilience and self-efficacy and their quality of life. Patient Prefer Adherence.

[R6] Wren AA, Ross AC, D'Souza G, Almgren C, Feinstein A, Marshall A, et al (2019). Multidisciplinary pain management for pediatric patients with acute and chronic pain: a foundational treatment approach when prescribing opioids. Children (Basel).

[R7] Bucsea O, Riddell RP (2019). Non-pharmacological pain management in the neonatal intensive care unit: Managing neonatal pain without drugs. Semin Fetal Neonatal Med.

[R8] Mohammadi F, Farjam M, Gholampour Y, Sohrabpour M, Oshvandi K, Bijani M (2021). Caregivers' perception of the caring challenges in coronavirus crisis (COVID-19): a qualitative study. BMC Nurs.

[R9] Vahdatpour B, Haghighat S, Sadri L, Taghian M, Sadri S (2022). Effects of Transfer Energy Capacitive and Resistive On Musculoskeletal Pain: A Systematic Review and Meta-Analysis. Galen Medical Journal.

[R10] Mohammadi F, Tehranineshat B, Bijani M, Khaleghi AA (2021). Management of COVID-19-related challenges faced by EMS personnel: a qualitative study. BMC Emerg Med.

[R11] Neshat H, Jebreili M, Seyyedrasouli A, Ghojazade M, Hosseini MB, Hamishehkar H (2016). Effects of Breast Milk and Vanilla Odors on Premature Neonate's Heart Rate and Blood Oxygen Saturation During and After Venipuncture. Pediatr Neonatol.

[R12] Soleymani B, Hemmati F, Pishva N (2019). Evaluation of the Effect of Oral Carnitine Supplementation on Apnea of prematurity in Premature Neonates in Hospitals Affiliated to Shiraz University of Medical Sciences. J Biochem Tech.

[R13] Eichenwald EC (2016). Apnea of prematurity. Pediatrics.

[R14] Schmidt B, Roberts RS, Davis P, Doyle LW, Barrington KJ, Ohlsson A, et al (2006). Caffeine therapy for apnea of prematurity. N Engl J Med.

[R15] Edraki M, Pourpulad H, Kargar M, Pishva N, Zare N, Montaseri H (2013). Olfactory stimulation by vanillin prevents apnea in premature newborn infants. Iran J Pediatr.

[R16] Henderson-Smart DJ, Steer PA (2001). Methylxanthine treatment for apnea in preterm infants. Cochrane Database Syst Rev.

[R17] Pillekamp F, Hermann C, Keller T, Von Gontard, Kribs A, Roth B (2007). Factors influencing apnea and bradycardia of prematurity-implications for neurodevelopment. Neonatology.

[R18] Marlier L, Gaugler C, Messer J (2005). Olfactory stimulation prevents apnea in premature newborns. Pediatrics.

[R19] Kanbur BN, Balci S (2020). Impact of the odors of vanilla extract and breast milk on the frequency of apnea in preterm neonates. Jpn J Nurs Sci.

[R20] Beker F, Opie G, Noble E, Jiang Y, Bloomfield FH (2017). Smell and taste to improve nutrition in very preterm infants: a randomized controlled pilot trial. Neonatology.

[R21] Yaghoubi S, Salmani N, Dehghani K, DavoodiZadehJolgeh H (2017). Investigating effect of olfactory stimulation by vanilla on the rate of apnea attacks in neonates with apnea of prematurity: A randomized clinical trial. Int J Pediatr.

[R22] Piri SM, Ghodsi Z, Shool S, Anjomshoa A, Azarhomayoun A, Jangholi E, et al (2021). Macrophage migration inhibitory factor as a therapeutic target after traumatic spinal cord injury: a systematic review. Eur Spine J.

[R23] Park YA, Im YJ (2020). The effects of a continuous olfactory stimulation using breast milk (COSB) on behavioral state and physiological responses in Korean premature infants. J Pediatr Nurs.

[R24] Alemdar DK, Ä°nal S (2020). The effect of individualized developmental care practices in preterm infants. Complement Med Res.

[R25] Aghagoli S, Salimi A, Salimi M, Ghazavi Z, Marofi M, Mohammadbeigi A (2016). Aromatherapy with rosa damascenes in apnea, bradycardia and Spo2 of preterm infants; a randomized clinical trial. Int J Pediatr.

[R26] Ataei Nakhaei, Javid A, Marefat M, Chaichy Z, Alshahrestani A, Nazarpour P (2019). Is Aromatherapy Effective for Apnea in Preterm Infants. A Systematic Review Int J Pediatr.

[R27] Gambari R (2011). Predictive Analyses of Biological Effects of Natural Products: From Plant Extracts to Biomolecular Laboratory and Computer Modeling. Evid Based Complement Alternat Med.

[R28] Kowalski J, Samojedny A, Paul M, Pietsz G, Wilczok T (2005). Effect of apigenin, kaempferol and resveratrol on the expression of interleukin-1beta and tumor necrosis factor-alpha genes in J774. 2 macrophages Pharmacol Rep.

[R29] Boskabady MH, Shafei MN, Saberi Z, Amini S (2011). Pharmacological Effects of Rosa Damascena. Iran J Basic Med Sci.

[R30] Marofi M, Sirousfard M, Moeini M, Ghanadi A (2015). Evaluation of the effect of aromatherapy with Rosa damascena Mill on postoperative pain intensity in hospitalized children in selected hospitals affiliated to Isfahan University of Medical Sciences in 2013: A randomized clinical trial. Iran J Nurs Midwifery Res.

[R31] Hongratanaworakit T (2009). Relaxing effect of rose oil on humans. Nat Prod Commun.

